# Age‐related changes in microRNAs expression in cruciate ligaments of wild‐stock house mice

**DOI:** 10.14814/phy2.15426

**Published:** 2022-08-22

**Authors:** Yalda A. Kharaz, Katarzyna Goljanek‐Whysall, Gareth Nye, Jane L. Hurst, Anne McArdle, Eithne J. Comerford

**Affiliations:** ^1^ Department of Musculoskeletal Ageing Sciences, Institute of Life Course and Medical Sciences University of Liverpool, William Duncan Building Liverpool UK; ^2^ The MRC‐Versus Arthritis Centre for Integrated Research into Musculoskeletal Ageing (CIMA) Liverpool UK; ^3^ School of Medicine IRC Laureate, Physiology, Human Biology Building, NUI Galway Galway Ireland; ^4^ Chester Medical School University of Chester Chester UK; ^5^ Institute of Infection, Veterinary and Ecological Sciences, Leahurst Campus University of Liverpool Neston UK

**Keywords:** ageing, cruciate ligaments, microRNA

## Abstract

Cruciate ligaments (CL) of the knee joint are injured following trauma or aging. MicroRNAs (miRs) are potential therapeutic targets in musculoskeletal disorders, but there is little known about the role of miRs and their expression ligaments during aging. This study aimed to (1) identify if mice with normal physical activity, wild‐stock house mice are an appropriate model to study age‐related changes in the knee joint and (2) investigate the expression of miRs in aging murine cruciate ligaments. Knee joints were collected from 6 and 24 months old C57BL/6 and wild‐stock house mice (*Mus musculus domesticus*) for ligament and cartilage (OARSI) histological analysis. Expression of miR targets in CLs was determined in 6‐, 12‐, 24‐, and 30‐month‐old wild‐stock house mice, followed by the analysis of predicted mRNA target genes and Ingenuity Pathway Analysis. Higher CL and knee OARSI histological scores were found in 24‐month‐old wild‐stock house mice compared with 6‐ and 24‐month‐old C57BL/6 and 6‐month‐old wild‐stock house mice (*p* < 0.05). miR‐29a and miR‐34a were upregulated in 30‐month‐old wild‐stock house mice in comparison with 6‐, 12‐, and 24‐month‐old wild‐stock house mice (*p* < 0.05). Ingenuity Pathway Analysis on miR‐29a and 34a targets was associated with inflammation through interleukins, TGFβ and Notch genes, and p53 signaling. Collagen type I alpha 1 chain (COL1A1) correlated negatively with both miR‐29a (*r* = −0.35) and miR‐34a (*r* = −0.33). The findings of this study support wild‐stock house mice as an appropriate aging model for the murine knee joint. This study also indicated that miR‐29a and miR‐34a may be potential regulators of COL1A1 gene expression in murine CLs.

## Graphical abstract

This study aimed to investigate age‐related changes in microRNAs expression in mouse cruciate ligaments. Our findings demonstrate that the miRNAs were differentially expressed in cruciate ligaments from aging mice and are suggestive of important regulators of the extracellular matrix of murine cruciate ligaments during aging.

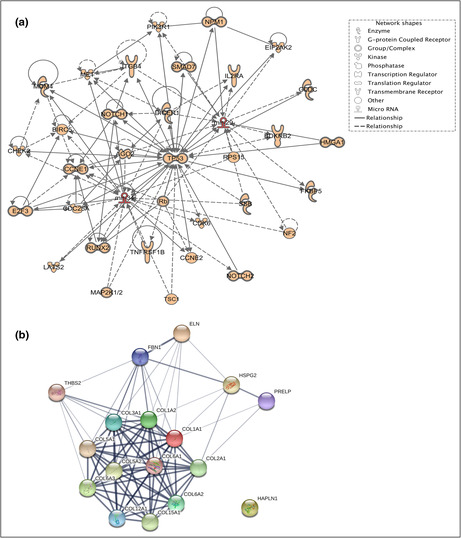

## 1 INTRODUCTION

The anterior and posterior cruciate ligaments (CLs) are the main stabilizers of the knee joint (Smith et al., 2011). Their composition consists of fibroblasts embedded in a specialized extracellular matrix (ECM), comprised mainly of type I collagen, and elastin with a range of noncollagenous proteins and proteoglycans (Kharaz et al., 2018). The knee joint anterior cruciate ligament (ACL) is one of the most frequently injured ligaments (Woo et al., 2000) resulting in significant joint instability, immobility (Woo et al., 2000), muscle atrophy (Mendias et al., 2013), and induction of knee joint osteoarthritis (OA) (Lohmander et al., 2007). Knee joint OA has major physical, social, and financial implications for the aging population (Cumps et al., 2008). ACL injury caused by trauma or contact sport only accounts for about 30% of ACL injuries (Cimino et al., 2010). The remaining ACL tears are from noncontact injuries, which can occur following gradual degeneration of the ligament ECM (Cimino et al., 2010). To date, the exact etiopathogenesis of noncontact ACL ruptures is not defined, however, risk factors have been identified such as age (Hasegawa et al., 2012), gender (Toth & Cordasco, 2000), body weight (Uhorchak et al., 2003), and genetics (Posthumus et al., 2009). The healing potential of the ACL is poor and reconstruction following traumatic injuries does not completely restore the functional stability of the knee joint (Gobbi et al., 2012), which contributes to the development of knee OA (Paschos, 2017). Therefore, more effective novel strategies for managing ACL injury should be developed to promote the healing of the native ligament structure to have a protective impact on the tissue mechanics and subsequently the articular cartilage of the knee (Shoji et al., 2012).

Aging has been shown to alter ligament ECM composition (Amiel et al., 1991; Comerford et al., 2006; Hasegawa et al., 2012, 2013). This has been demonstrated in aged humans and canine ACL, where changes in the ECM ultrastructure have been reported prior to cartilage injury and other signs of knee joint OA (Hasegawa et al., 2012, 2013). Amiel et al. (1991) demonstrated that collagen content and synthesis decreased with age in knee ligaments from rabbits, resulting in a significant effect on the mechanical properties of these tissues. ACLs from healthy aged dogs (and dog breeds at a high risk to ACL injury) show increased chondroid metaplasia of ligament fibroblasts and reduced cell density compared with ACLs from young and low‐risk animals (Comerford et al., 2006). Similar changes in degenerated ACLs of aged humans occur, with decreased fibroblast density being found in ACLs of older people (Hasegawa et al., 2013). To date, there are no treatment options targeting the prevention of ligament ECM degradation, which leads to ligament damage and eventual rupture in high disease risk species, such as dogs or men.

MicroRNAs (miRNAs or miRs) control the simultaneous expression of many genes and have been proposed as potential therapeutic molecules for disorders of musculoskeletal tissues such as ligaments due to their relatively easy delivery into tissues (Soriano‐Arroquia et al., 2016). They are small endogenous (~22 nt) noncoding RNAs that play important regulatory roles in animals and plants through posttranscriptional modulation of gene expression by binding and repressing the expression of specific mRNAs (Bartel, 2004). miRNAs have been demonstrated to play a role in disease repair mechanisms in a number of different tissues, including tendons and ligaments (Li et al., 2017; Millar et al., 2015; Shoji et al., 2012). A recent study in human ACLs has demonstrated 39 differentially expressed miRs between patients with and without knee OA. Twenty‐two miRs, such as 26b‐5p and 146a‐5p, were found to be upregulated, whereas 17 miRs, such as 18a‐3p and 138‐5p, were downregulated in the osteoarthritic ACL tissues, suggesting that related miRNA dysregulation is involved in ligament injury in patients with OA (Li et al., 2017). Another study demonstrated that miR‐210 was decreased in partially transected ACLs in a rat model suggesting that miR‐210 promotes ACL healing through the enhancement of angiogenesis (Shoji et al., 2012). To date, there is little known about the role of miRNAs and their expression in ACLs during aging and limited data on their use in either treating or preventing ligament injuries.

There are currently numerous rodent models that are used in orthopedic research, including mice, rats, gerbils, and squirrels, among others; with mice and rats being the more frequently used models (Allen et al., 2017; Iannaccone & Jacob, 2009). Laboratory mice such as the C57BL/6 strain have been domesticated, possibly over a period of up to 3000 years (Ferris et al., 1982) and show considerably reduced activity and speed of movement compared with wild house mice due to both genetic changes and substantial environmental constraints (Yang et al., 2007). In comparison with C57BL/6 mice, an inbred strain of house mice (*Mus musculus domesticus*) derived from the wild in 1978 has been found to exhibit considerably greater activity under standard caged conditions (Nishi et al., 2010), suggesting that laboratory animals more recently derived from the wild may provide a more appropriate model to study age‐related changes associated with normal physical activity. In this study, we hypothesized that (1) wild‐stock mice are an appropriate model in comparison with C57BL/6 mice to study age‐related changes in the knee joint associated with normal day‐to‐day activities and (2) there are age‐related changes in miRNAs expression in CLs of mice associated with normal physical activity. Therefore, this study aimed to compare age‐related changes between wild‐stock house mice and C57BL/6 using histological analysis and to investigate miRNAs expression in cruciate ligaments during aging of mice associated with normal physical activity.

## 2 METHODS

### 2.1 Animals

Six‐ and 24‐month‐old specific pathogen‐free (SPF) C57BL/6 mice were purchased from Charles River (Lyon, France) and delivered to the Biomedical Services Unit at the University of Liverpool at least 4 weeks prior to the required age to allow acclimation. Mice were individually housed and were fed a CRM (P) rodent diet with ad libitum access to food and were maintained under barrier conditions in microisolator cages on a 12‐h dark/light cycle.

Wild‐stock house mice (*Mus musculus domesticus*) were originally derived from local Cheshire populations, outbred in captivity for 1–4 generations (Ramm et al., 2015). Animal use and care was in accordance with EU directive 2010/63/EU and UK Home Office code of practice for the housing and care of animals bred, supplied or used for scientific purposes. The University of Liverpool Animal Welfare Committee approved the maintenance of our wild mouse colony, but no specific licenses were required. These mice were euthanized for reasons unrelated to this study and the knee joint tissue was obtained postmortem as clinical waste. Mice were fed with Corn Cob Absorb 10/14 substrate (IPS Product Supplies Ltd) and with ad libitum access to food (LabDiet 5002, Purina Mills) and water. All wild‐stock mice were provided with paper wool nesting materials (IPS Supplies Ltd) and a variety of cardboard tubes or boxes and plastic tubes or clip‐on shelters hanging from the cage top. Subjects were maintained under controlled environmental conditions as follows: temperature 20–21°C, relative humidity 45%–65%, and a reversed 12:12 h light cycle.

### 2.2 Tissue collection

The entire knee joint from 6‐ and 24‐month‐old C57BL/6 and wild‐stock house mice (*n* = 6) were collected and stored in 4% paraformaldehyde for 48 h for histological analysis. For RNA extraction, ACLs and posterior cruciate ligaments (PCLs) were isolated with a dissecting microscope (Olympus CK40) from 6‐, 12‐, 24‐, and 30‐month‐old wild‐stock house mice, snap frozen, and stored at −80°C until required.

### 2.3 Knee joint collection and histological analysis

Following fixation, knee joints were decalcified for 4 weeks in a solution of 25 g EDTA in 175 cm^3^ distilled water (pH = 4–4.5), wax‐embedded, and 6 μm coronal sections cut through the whole joint (Javaheri et al., 2016). Sections were scored twice by one observer blinded to the sample origins using the OARSI grading system for mouse knee joint cartilage (Glasson et al., 2010) and using a comparative grading system for ligament scoring (Table 1). Lesion severity in articular cartilage in these joints was graded from 0 to 6 based on the extent of changes (Table 1). The four compartments of the tibiofemoral joint were graded throughout the entire joint allowing the determination of maximum lesion grade for the whole joint. The mean score for each joint was determined by calculating the average grade across multiple slides (Poulet et al., 2011).

**TABLE 1:** Histological scoring criteria for cruciate ligament and OARSI

	Parameter
Cruciate ligament scoring	ECM staining
	Cell hypertrophy
	Cell clustering
	Loss of alignment
Cartilage degeneration scoring	Normal articular cartilage, no degeneration (grade 0)
	Loss of Safranin‐O without structural changes (grade 0.5)
	Small fibrillations without loss of cartilage (grade 1)
	Vertical clefts down to the layer immediately below the superficial layer and some loss of surface lamina (grade 2)
	Vertical clefts/erosion to the calcified cartilage extending to <25% of the articular surface (grade 3)
	Vertical clefts/erosion to the calcified cartilage extending to 25–50% of the articular surface (grade 4)
	Vertical clefts/erosion to the calcified cartilage extending to 50–75% of the articular surface (grade 5)
	Vertical clefts/erosion to the calcified cartilage extending >75% of the articular surface (grade 6)

Both ACLs and PCLs were scored based on strength of ECM staining, and intensified red coloration indicates an increase in proteoglycans, cell hypertrophy, abnormal increase in cell size, cell clustering loss, the disorganization of spindle‐shaped flattened cells, and predominant fibers and loss of alignment of collagen fibers. Each parameter was graded from 0 to 4 based on the extent of changes (0 = normal, 0% increased; 1 = mild abnormality, 5%–25% increase; 2 = moderate abnormality, 26%–50% increase; 3 = marked abnormality, 51%–75% increase; and 4 = severe abnormality, 76%–100% increase) (Kharaz et al., 2018, 2021).

### 2.4 MicroRNA: Target interaction prediction

Experimentally predicted miRNAs from previously identified ECM proteins/gene in ligaments (Kharaz et al., 2016) were determined using Targetscan Human (Version 7.1) as described previously (Soriano‐Arroquia et al., 2016) (Table 2). MicroRNAs predicted to regulate several ECM components of ligaments were investigated in order to identify miRs that regulate ligament programs, pathways, and networks rather than individual genes.

**TABLE 2:** Identification of predicted biological targets of microRNA (miR) in murine cruciate ligaments through TargetScan

miRs	Predicted ECM gene targets ID
miR‐1	COL5A2, FMOD, HAPLN1, FBLN2, THBS2, TNMD
miR‐7	COL2A1, COL5A2, COL14A1, KERA, CILP1, FBLN1, MFAP5
miR‐9	COL1A2, COL5A1, COL12A1, COL15A1, ASPN,FBN1, FBN2, TNN, THBS2
miR‐15	COL12A1, PRELP, HSPG2, MFAP5, TNMD, LUM, THBS2
miR‐19	TNN, THBS1, CILP2
miR‐17	TNC, THBS2
miR‐21	COL12A1, ASPN, LUM, FBN1, MATN2, THBS2, THBS3
miR‐22	HSPG2, OGN
miR‐23	COL5A2, COL6A1, COL6A3, COL14A1, COL15A1, ASPN, FMOD, LUM, VCAN, FBN1, FBN2, MFAP5, THBS1
miR‐24	THBS4
miR‐25	ACAN, PRELP, CHAD, CILP2, FBN1, FBLN1
miR‐27	COL1A2, COL12A1, ACAN, ASPN, LUM, FBN1, FBN2, FBN2, MATN2
miR‐29	COL5A1, HAPLN1, VCAN, FN1, FBN2, FBLN2, THBS1
	COL1A1, COL1A2, COL2A1, COL3A1, COL5A1, COL5A2, COL6A1, COL6A2, COL6A3, COL15A1, PRELP
miR‐30	HSPG2, HAPLN1, FBN1, THBS2
miR‐31	COL12A1, COL14A1, TNXB
miR‐34	COL5A1, FMOD, PRELP, FBLN1
miR‐96	COL1A1, COL5A1, COL5A2, COL12A1, PRELP, ELN
miR‐101	COL1A1, COL5A1, ACAN, ASPN, DCN, VCAN, FBLN1, THBS1
miR‐103	COL5A1, COL12A1, ASPN, BGN, VCAN, HAPLN1, FBN1, FBN2, FN1, THBS1, THBS4
miR‐122	COL6A1, COL6A3
miR‐124	BGN, CHAD, FMOD
miR‐125	COL6A3, COL12A1, ACAN, VCAN, FMOD, PRELP, CILP1, ELN, MATN2, THBS2, TNMD
miR‐128	COL5A1, FN1, HSPG2, CILP2
mir‐129	COL3A1, COL5A1, FMOD, HAPLN1, FN1, FBLN2, LUM, VCAN, ELN, TNXB
miR‐130	COL1A1, ACAN, FMOD, OGN, PRELP, VCAN, COMP
miR‐ 133	COL6A3, HAPLN1, CILP1, MFAP5, TNN
miR‐140	COL1A2, COL6A3, ACAN, HAPLN1, CILP2, FBN2, ELN, THBS1, TNN
	COL5A1, COL5A2, COL6A3, HSPG2, OGN, PRELP, CILP1, FN1, FBN1, FBN2, FBLN1, FBLN2, MATN2, TNN
miR‐142	THBS1, THBS2, THBS4
miR‐145	DCN, KERA, PRG4
miR‐143	DCN, FN1, CILP, TNN
miR‐148	COL1A1, COL5A1, COL14A1, PRELP, FN1, MATN2, THBS1
miR‐150	COL2A1, COL6A1, COL6A3
miR‐155	BGN, PRELP, TNC
miR‐181	KERA, OGN, VCAN, TNMD, THBS1, THBS2, FBLN1, TNMD
miR‐196	COL5A1, COL6A3, ACAN, ASPN, HAPLN1, OGN,DCN, CILP1, FBN1, FBN2, THBS1, THBS2, THBS4
miR‐199	COL1A1, COL3A1,COL14A1, CILP2
miR‐203	CHAD, HAPLN1, CILP1, FN1, MATN2, TNC, TNMD, THBS2, MATN2
miR‐205	ACAN, ASPN, FMOD, DCN, FBN1, MFAP5, TNC
miR‐214	COL14A1
miR‐218	COL1A1, HSPG2, ELN, TNMD
miR‐219	PRG4, TNN
miR‐455	COL15A1, ASPN, CHAD, KERA, PRG4, CILP2, THBS4
	COL2A1, COL6A1, COL12A1, COL14A1, COL15A1, ACAN, ASPN, DCN, HSPG2 KERA, PRG4, OGN, PRELP
miR‐489	VCAN, CILP2, ELN, TNC, THBS1
miR‐499	COL1A2, DCN, HAPLN1, CILP2, FBN1
miR‐503	COL5A2, COL14A1, VCAN, TNN, MATN2, FBN1, FBN2
Let‐7	COL6A1, COL6A2, LUM
	COL1A1, COL1A2, COL3A1, COL5A2,COL14A1, COL15A1, DCN, THBS1

### 2.5 Interaction network analysis

Pathway analysis of differentially expressed miRNAs was produced using QIAGEN Ingenuity Pathway Analysis (IPA) Product (Ingenuity_Systems, http://www.ingenuity.com), the Core Analysis function, and the Path Designer feature (System I, 2014). Network interaction maps of predicted ECM target genes were created by the differently expressed miRNA using the String bioinformatics tool (String‐DB) version 9.1 by allowing for experimental evidence in addition to the predicted functional links: co‐occurrence, co‐expression, databases, and text‐mining (Franceschini et al., 2013).

### 2.6 RNA isolation and real‐time PCR

Total RNA isolation was performed using standard methods as described previously (Goljanek‐Whysall et al., 2014). Details of primers are included in Tables S1 and S2. In brief, RNA was extracted with Trizol (Invitrogen™ Life Technologies), quantified according to the manufacturer's protocol using a Nanodrop ND‐100 spectrophotometer (Labtech), and assessed for purity by UV absorbance measurements at 260 and 280 nm. cDNA synthesis for miRNA was performed using 200 ng RNA and miRscript RT kit II (Qiagen) according to the manufacturer's protocol. MicroRNA qPCR analysis was performed using miRScript Sybr Green Mastermix (Qiagen) in a 20 μl reaction (Applied Biosystems 7500 Fast real‐time PCR system) following the manufacturer's protocols. The qPCR conditions were 95°C for 15 min for initial activation followed by 40 cycles of 95°C for 30 s, 55°C for 30 s, and 70°C for 30 s.

cDNA synthesis for gene expression analyses was performed using 500 ng RNA using Moloney murine leukemia virus reverse transcriptase and random hexamer oligonucleotide (both from Promega) using 500 ng RNA in a 25 μl reaction. mRNA qPCR was performed on 5 μl 10× diluted cDNA by employing a final concentration of 300 nM of each primer in a 20 μl reaction on the ABI 7700 sequence detector using MESA Blue SYBR Green reagent (Eurogentec) using the following protocol: denaturation at 95°C for 5 min, followed by 40 cycles of DNA amplification (15 s 95°C and 45 s annealing at 60°C) (Peffers et al., 2015). Used miRNA primers in this study have been validated in previous publications (Soriano‐Arroquia et al., 2016) and supplied by Eurogentec (Table S1). mRNA and miRNA data sets were compared with the designated control utilizing housekeeping genes *GAPDH* or *RnU6* as detailed in figure legends.

### 2.7 Statistical analysis

Statistical analysis was performed for both histological scoring and qRT‐PCR data using Graphpad Prism (Version 7, GraphPad Software) and set at a significance level of 5%. The normal distribution for each data set was assessed using a Kolmogorov–Smirnov test and a one‐way ANOVA with a Tukey post hoc test comparing histological scoring between C57BL/6 and wild‐stock house mice.

Intraobserver agreement of histological scoring systems was calculated using Cohen's kappa coefficient (www.statstodo.com/CohenKappa_Pgm.phpl). One‐way ANOVAs with Tukey post hoc test were assessed for differences in qRT‐PCR data between age groups. Pearson's correlations (*r*) assessed relationships between differentially expressed miRNA and mRNA target genes.

## 3 RESULTS

### 3.1 Morphological differences between the wild‐stock house and C57BL/6 mice

**FIGURE 1:**
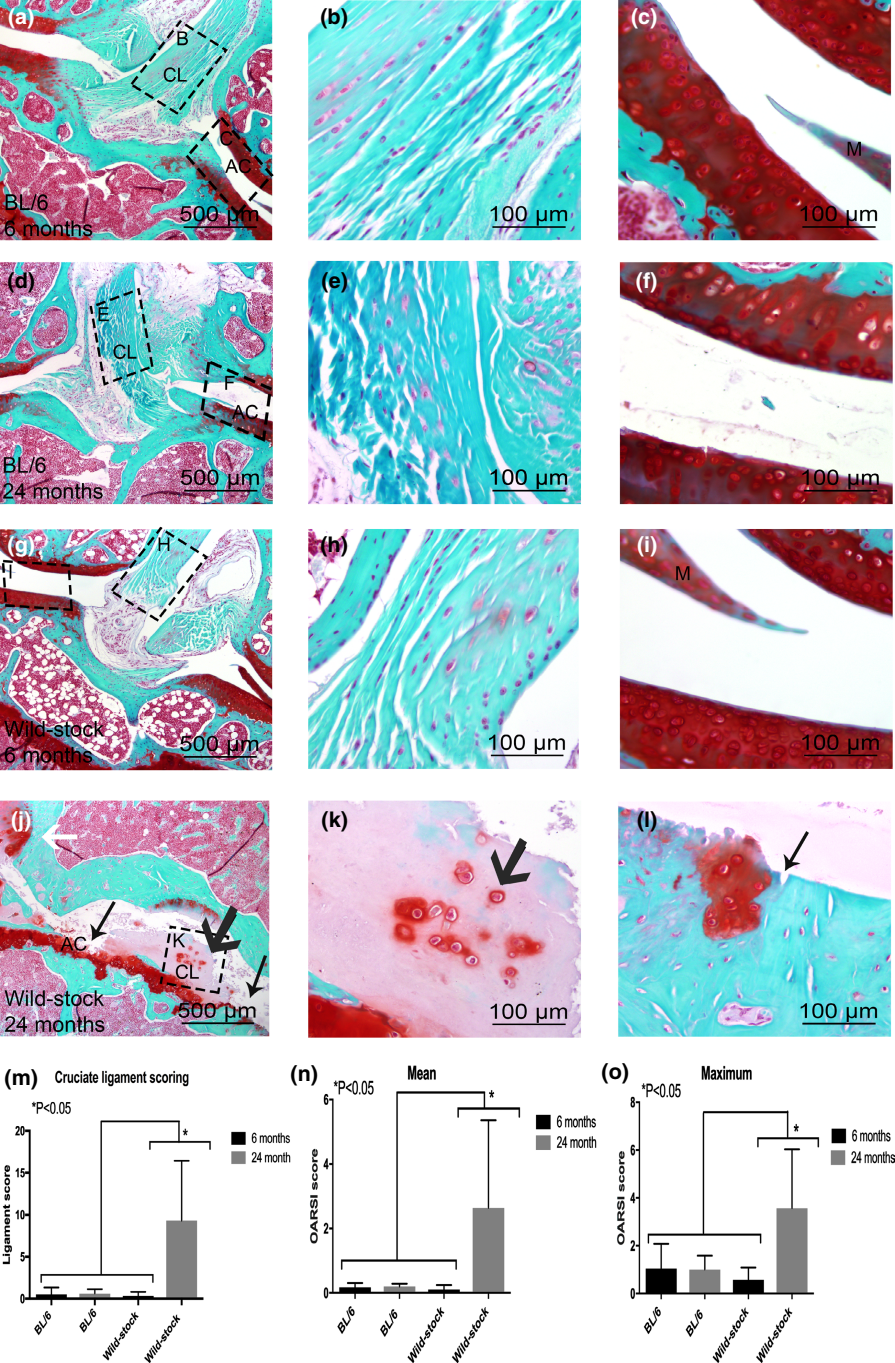
Histological comparison between 6‐month‐old C57BL/6 (a–c), 6‐month‐old wild‐stock house mouse (d–f), 24‐month‐old C57BL/6 (g–i), and 24‐month‐old wild‐stock house (j–k) mouse with Safranin‐O knee joints. On histological images cruciate ligament (CL), articular cartilage (AC), and meniscus (M) is shown. An abnormal structure of cruciate ligaments with chondrocytic cell morphology and increased proteoglycan content around cells was observed (black wide arrow in j and k). Erosion to the calcified cartilage extending >75% of the articular surface (black arrow in j and l) and osteophyte formation and structural changes in meniscus were also observed (white arrows in j). Statistically significantly higher CL and OARSI scores were measured in 24‐month‐old wild‐stock house mice compared with 6‐, 24‐month‐old C57/BL6, and 6‐month‐old wild‐stock house mice (m–o). Data are means ± SEM.

An overall Kappa statistic of 0.8 for blinded intraobserver agreement for the histological grading was calculated, indicating strong agreement. Histological observation showed no changes between 6 months C57BL/6 (Figure 1A–C) 24 months C57BL/6 (Figure 1D–F) and 6‐month‐old wild‐stock house mice (G‐I). Furthermore, there were no statistically significant differences found in the CL and OARSI scores within these groups.

Increased cell hypertrophy, clustering, loss of collagen architecture, and ECM staining (proteoglycan) content around cells were observed in 24‐month‐old wild‐stock house mice (Figure 1J,K bold black arrows).

Twenty‐four‐month‐old wild‐stock house mice also showed severe cartilage lesions extending >75% of the articular surface observed (Figure 1L, narrow black arrows). In addition, osteophyte formation was also observed (Figure 1K, white arrows). Both the CL (Figure 1M) and OARSI mean and maximum scores (Figure 1N,O) were significantly higher in 24‐month‐old wild‐stock house mice (*p* < 0.05) in comparison with 6‐month‐old wild‐stock house mice as well as 6‐ and 24‐month‐old C57BL/6 mice.

### 3.2 MicroRNA expression and IPA

**FIGURE 2:**
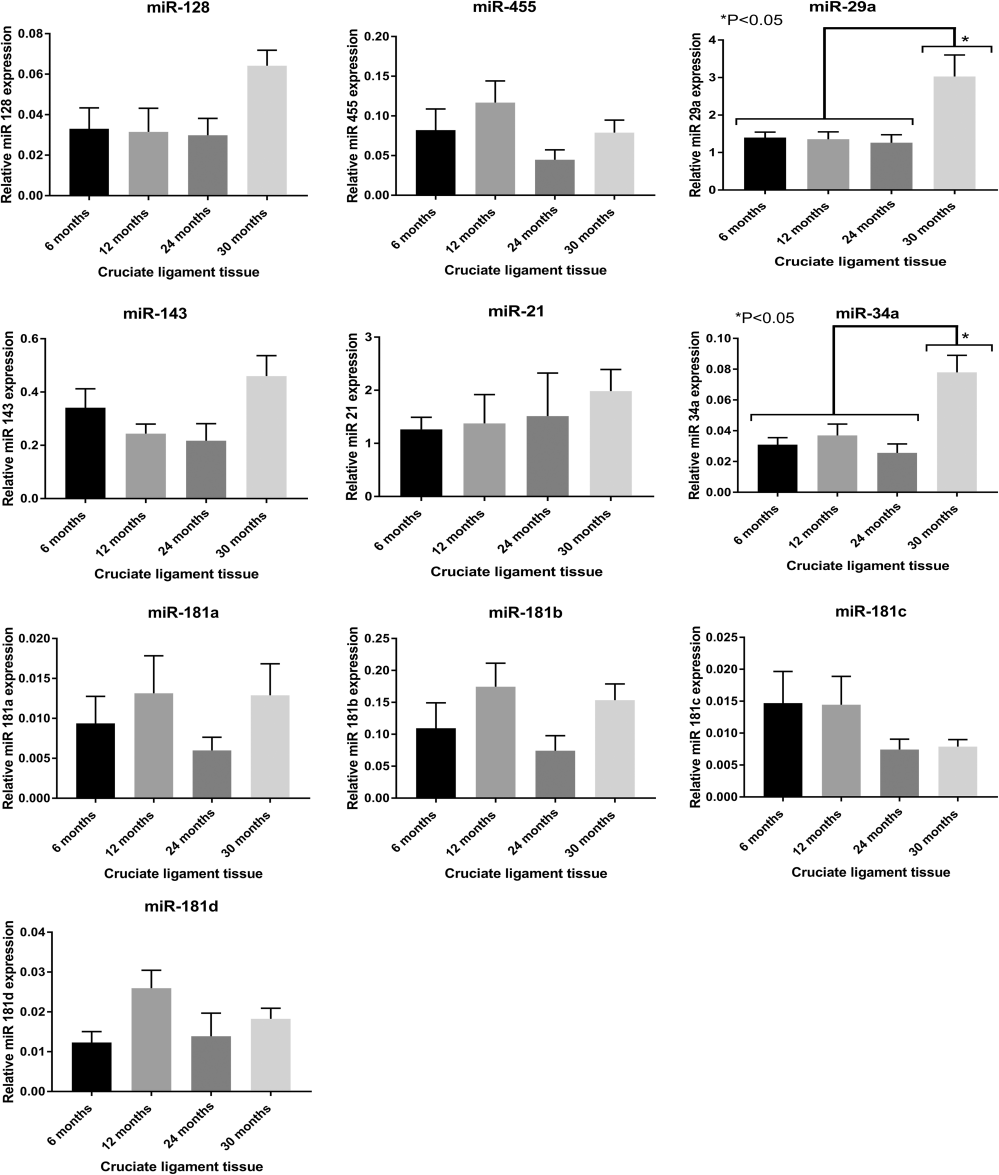
MicroRNA (miR) relative expression (to housekeeping gene *Rnu6*) at different ages in murine cruciate ligaments. Statistically significantly higher expression of miR‐29a and miR‐34a was found in 30‐month‐old mice in comparison with the 6‐, 12‐, and 24‐month‐old mice. Data are means ± SEM.

**FIGURE 3:**
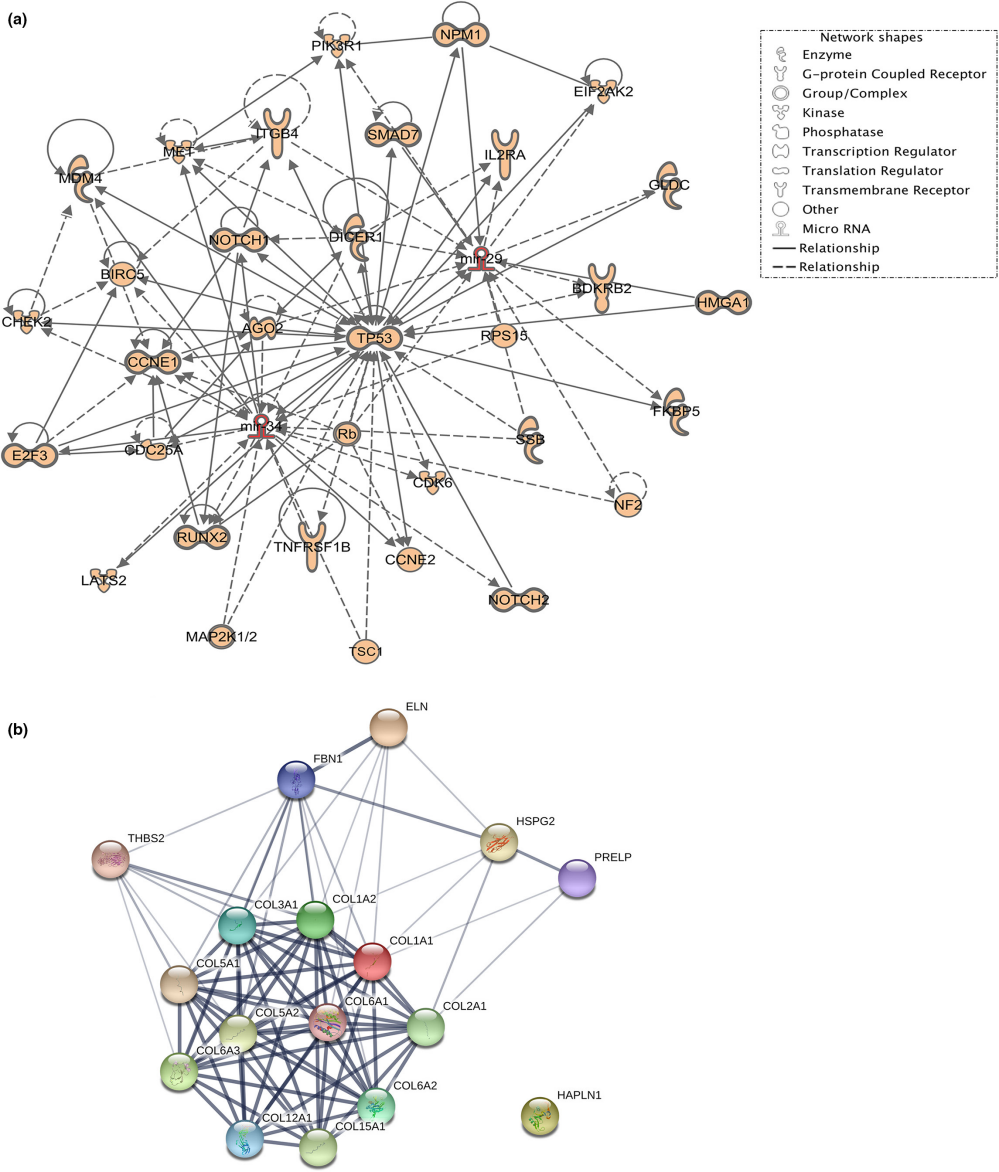
(a) Network of interactions constructed for the microRNA targets (miR‐29a and miR‐34a) using ingenuity pathway analysis software. (b) An interaction map of extracellular matrix target genes predicted through target scan to be upregulated by both miR‐29a and miR‐34a was built with STRING. One highly connected cluster was evident around collagen proteins, and a high confidence level (0.0700) was allowed for experimentally predicted gene–gene interaction.

The miRNA expression was only performed in 6‐, 12‐, 24‐, and 30‐month‐old wild‐stock house mice. The expression of several miRNAs that were predicted through Target Scan (Table 1) to regulate key ECM components associated with ligament aging was determined. There were no significant differences in expression levels between any of the age groups with miR‐128, miR‐455, miR‐143, miR‐21, miR‐34a, and miR‐181 (Figure 2). However, miR‐29a and miR‐34a were expressed at significantly higher levels in 30‐month‐old mice (*p* < 0.05) in comparison with the 6‐, 12‐, and 24‐month‐old mice (Figure 2). IPA indicated that upregulation of miR‐29a and miR‐34a during aging in CLs may be associated with mitogen‐activated protein kinase (MAPK), p53, SMAD and Notch signaling, as well as inflammation‐related genes such as TGFβ (transforming growth factor beta) and interleukin‐2 (Figure 3A). String‐DB analyses of ECM‐associated predicted target genes of miR‐29a and miR‐34a revealed a network of genes containing one highly connected cluster around collagens ECM organization (Figure 3B). The two significant Kegg pathways identified by String were ECM receptor interaction and focal adhesion (Bonferroni adjusted *p* values of 1.81e‐13 and 5.94e‐09, respectively).

### 3.3 Gene expression of target genes

**FIGURE 4:**
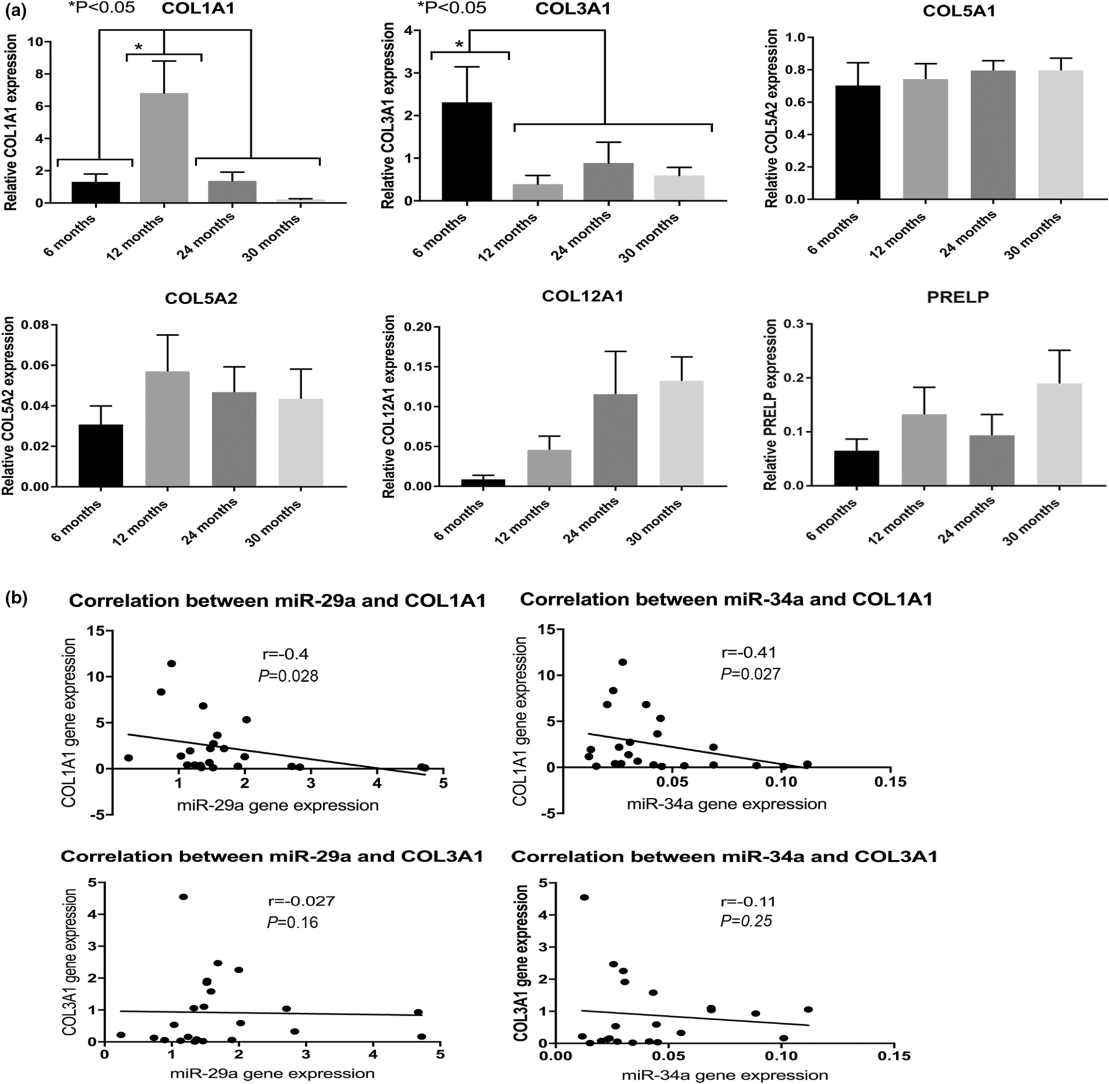
(a) Relative expression (to housekeeping gene *GAPDH*) of extracellular matrix predicted mRNA target genes for miR‐29a and miR‐34a in mice cruciate ligament. (b) Pearson's correlation demonstrated statistically significant negative correlations between miR‐29a and miR‐34 with COL1A1 target gene (*r* = −0.41, *p* < 0.05 and *r* = −0.4, *p* < 0.05). Pearson's correlation between miR‐29a and miR‐34a with COL3A1 was not found to be statistically significant. Data are means ± SEM.

Target gene expression of several ECM‐associated targets predicted to be regulated for both miR‐29a and miR‐34a using TargetScan and Ingenuity Pathway Analyses was measured. These mRNA targets were also identified previously in nondiseased canine ACLs as ligament markers (Kharaz et al., 2016). Measurement of mRNA targets included collagen type I alpha‐1 chain (COL1A1), collagen type 3 alpha‐1 chain (COL3A1), collagen type V alpha‐1 chain, collagen type XII alpha‐1 chain (COL12A1), and proline‐ and arginine‐rich end leucine‐rich repeat protein (PRELP) (Figure 4A). The expression of COL1A1 was significantly higher in CLs of mice at 12 months (*p* < 0.05) compared with 6‐, 24‐, and 30‐month‐old mice (Figure 4A). Significantly higher expression of COL3A1 (*p* < 0.05) was also measured in 6‐month‐old mice than 12‐, 24‐, and 30‐month‐old mice (Figure 4A). Pearson's correlation analyses demonstrated statistically significant negative correlations between miR‐29a and miR‐34a and COL1A1 expression levels (*r* = −0.41 and −0.4) (*p* < 0.05) (Figure 4B). Pearson's correlation between the expression of miR‐29a and miR‐34a with COL3A1 (*r* = −0.03 and −0.11) was not statistically significant (Figure 4B).

## 4 DISCUSSION

This study has identified age‐related morphological differences in the knee joints and CLs from wild‐stock house mice that exhibit normal physical activity, in contrast to no effect of age seen in joints and ligaments of C57Bl/6 mice. Wild‐stock house mice may therefore be a more appropriate model of healthy aging compared with joints and ligaments of C57BL/6 mice. This is the first study to measure the differential microRNA gene expression in murine cruciate ligaments with age. Our findings demonstrate that the miRNAs (miR‐29a and miR‐34a) were differentially expressed in cruciate ligaments of 30‐month‐old wild‐stock house mice. Both miR‐29a and miR‐34a negatively correlated with COL1A1 gene expression, suggestive of their role as potential regulators of the ECM of murine CLs during aging.

Our histological analysis of the knee joints in different age groups of C57BL/6 and wild‐stock house mice demonstrated morphological changes in tissue structures, such as the formation of osteophytes and changes in CL structure along with significantly higher histology scores in knee joint femoral and tibial cartilage, in the aged wild‐stock mice compared with 6‐month‐old wild‐stock house mice and age‐matched C57Bl/6 mice. These findings suggest that wild‐stock house mice are a more appropriate aging model to study ligament degeneration, and so were further used to investigate the regulation of miRNAs in aging CL.

Among a subset of miRs, miR‐29a and miR‐34a were found to have a significantly higher expression in 30‐month‐old mice in comparison with the other age groups of wild‐stock house mice. Both miR‐29a and miR‐34a have been found to regulate age‐related diseases such as vascular aging (Boon & Dimmeler, 2011) and cellular senescence in muscle (Hu et al., 2014). Studies into OA pathogenesis in human and mouse cartilage have also demonstrated upregulation of both miR‐29a (Le et al., 2016) and miR‐34 (Yan et al., 2016). In the current study, the differential expression of miRs measured in the aging murine CLs may be associated with the knee joint cartilage erosion and OA pathogenesis of these aging mice, as observed through our histological staining. Together, these findings may indicate that the upregulation of miR‐29a and miR‐34a in aging murine CLs could be associated with late‐stage pathology of OA and may be key to later stages of joint degeneration.

During aging, signaling pathways, including TGFβ, NOTCH, pSMAD, IGF, and MAPK, are known to play a role in tissues such as mouse and human skeletal muscle (Carlson et al., 2008; Williamson et al., 2003) and knee joint cartilage (Xu et al., 2016). In the ACL, the exact signaling pathway mechanism is yet to be elucidated during aging and disease. However, our constructed predicted network using IPA contained signaling pathways such as TGFβ and MAPK, p53, miR‐29a, and miR‐34a. miR‐29 has also been found to activate p53 in murine fibroblasts through repression of Ppm1d phosphatase during aging and DNA damage activation (Ugalde et al., 2011). Together, these data demonstrate the potential signaling networks with mice cruciate aging and the association of miRs. Further functional investigation is required to study the association networks between miRs regulation and signaling pathways during CL aging.

In the current study, both miR‐29a and miR‐34a were predicted to target several collagen genes including type I, II, III, V, VI, and XII, which were highly connected as cluster interaction identified by String analysis. Other ECM genes such as the PRELP, HAPLN1, HSPG2, FBN1, and THBS2 were also predicted as target genes. We primarily focused on the validation of several target genes that have previously been identified as ligament ECM genes and are widely expressed (Kharaz et al., 2016, 2018; Little et al., 2014) and particularly on target genes COL1A1 and COL3A1 genes, where higher mRNA levels have been found in ruptured compared with healthy ACLs (Lo et al., 1998; Young et al., 2011). We found significant alterations in mRNA expression levels of collagen types I and III during aging of murine CLs. The reduced expression of COL3A1 levels in the current study in mice after 6 months of age does not agree with studies of equine superficial digital flexor tendon, where higher levels of COL3A1 were found with aging (Birch et al., 1999). This may suggest differences between COL3A1 turnover rates between species and tissue types. The increased expression of COL1A1 levels at 6 months of age followed by a decreased expression in aging mice may be due to dynamic interactions and feedback mechanisms between miRNA and targets genes. Nevertheless, changes in the mRNA levels of collagens need to be considered carefully, given the posttranslational modification of these proteins affecting their function.

Apart from miR‐29a and miR‐34a, other miRs such as miR‐133a (Castoldi et al., 2012) and miR‐129 (Chen et al., 2018) have also been demonstrated to regulate COL1A1 gene expression in cardiac and liver tissues but may also be involved in the aging cruciate ligament in the current study. The decreased expression of COL1A1 in mice 12 months of age in our study may also have contributed to the abnormal structure of the CLs found in our histological analysis. Previous studies have demonstrated changes in aging equine tendons in their collagen cross‐link profile following glycation (Thorpe et al., 2010) and an increase in collagen degradation markers (Thorpe et al., 2010), which may also agree with the abnormal ECM structure and the decreased levels of COL1A1 in the aging mice CL in this study. Further studies are required to measure the accumulation of damage in mice CL collagen during aging.

We found negative correlations primarily between COL1A1 gene expression and miR‐29a and miR‐34a, suggestive of potential regulation of this vital gene in CL tissue pathology during aging. In tendon injuries, miR‐29a has been demonstrated as a posttranscriptional regulator of collagen type III gene expression in murine and human tendon injury, through interleukin‐L33 (Millar et al., 2015). However, we did not find any statistically significant negative correlations between either miR‐29a and miR‐34 and COL3A1, suggesting that miR‐29a may play different roles in the aging versus injury processes. Future studies will involve in vitro miRNA target validation and in vivo delivery of miR‐29a and miR‐34a in wild‐stock mice knee joints followed by phenotypic analyses.

One limitation of this study is that it was underpowered primarily due to availability of wild‐stock house mice and would benefit from further histological analysis of 30‐month‐old wild‐stock house mice for assessment of morphological features. Another limitation was the lack of ligament‐specific and ACL‐specific matrix markers for the validation of target genes. Future research should focus on validation markers in healthy and diseased cruciate ligaments as this will provide us insight into the understanding of miRNA expression and ligament disease pathways.

In conclusion, CL aging and degeneration, resulting in injury can have severe physical, social, and economic consequences on the affected individual and will lead to the development of degenerative joint diseases such as OA. Through our histological analysis, we have shown that wild‐stock house mice are an appropriate mouse model to study spontaneous age‐related changes in the knee joint compared with C57BL/6 mice. This study also indicated that miR‐29a and miR‐34a may be potential regulators of COL1A1 gene expression in CLs, possibly associated with an ultrastructural deterioration of these tissues during aging. These miRNA–gene target interactions may then be responsible for age‐related pathophysiological processes in CLs through regulation of inflammatory‐related genes, Notch signaling, MAPK, and p53 signaling.

## AUTHOR CONTRIBUTIONS

YAK, KW, GN, JH, AM, and EC contributed to the conception and design of the work. YAK, KW, JH, and EC performed the experiments. YAK analyzed the data. YAK, KW, AM, and EC drafted the initial manuscript. All authors have read, revised, and approved the manuscript.

## FUNDING INFORMATION

This study was funded in part by BBSRC (BB/L021668/1). We would like to thank the research support budget from the Department of Musculoskeletal Aging (Institute of Aging and Chronic Disease) for the funding provided for this study.

## CONFLICT OF INTEREST

The authors declare that they have no conflict of interest.

## ETHICS STATEMENT

Procedures were performed in accordance with UK Home office Guidelines under the UK Animal (Scientific Procedures Act 1986) and received ethical approval from the University of Liverpool Animal Welfare Committee.

## Supporting information


TableS1‐S2
