# Correction to “Age‐related changes in microRNAs expression in cruciate ligaments of wild‐stock house mice”

**DOI:** 10.14814/phy2.70822

**Published:** 2026-03-17

Kharaz, Y. A., Goljanek‐Whysall, K., Nye, G., Hurst, J., McArdle, A., & Comerford, E. J. (2022). Age‐related changes in microRNAs expression in cruciate ligaments of wild‐stock house mice. *Physiological Reports*, 10, e15426. https://doi.org/10.14814/phy2.15426.

The American Physiological Society and The Physiological Society (UK) are issuing a Society Note to include an Editor Disclosure Statement that was accidentally omitted from this published article in *Physiological Reports*. It should have been part of the Conflict of Interest statement.

## EDITOR DISCLOSURE STATEMENT

Anne McArdle, a coauthor on this article, was an Associate Editor of the journal at the time the article was published. The Societies verify that Anne McArdle was not involved in the handling of the manuscript and did not have access to information regarding the peer‐review process or final disposition of the article. An alternate editor oversaw the peer‐review and decision‐making processes.

