# Supplementary material for: Age‐related changes in microRNAs expression in cruciate ligaments of wild‐stock house mice
Source: Physiol Rep. 2022 Aug 22;10(16):e15426. doi: 10.14814/phy2.15426 (PMC9393909; doi:10.14814/phy2.15426)
Supplement: Supplementary file 1 — TableS1‐S2 [file PHY2-10-e15426-s001.docx]

Supplementary Tables:

Table 1: List of used microRNA primers as below

| **Product** | **Product number** | **Source** |
| --- | --- | --- |
| miRScript RT II | 218161 | Qiagen |
| miRScript SybrGreen | 218073 | Qiagen |
| RNU-6 qPCR primer | MS00033740 | Qiagen |
| miR-128 primer | MS00008582 | Qiagen |
| miR-455 primer | MS00009744 | Qiagen |
| miR-29a primer | MS00003262 | Qiagen |
| miR-143 primer | MI0000459 | Qiagen |
| miR-21 primer | MI0000077 | Qiagen |
| miR-34a primer | MI0000268 | Qiagen |
| miR-181a primer | MS00011263 | Qiagen |
| miR-181b primer | MS00006699 | Qiagen |
| miR-181c primer | MS00008841 | Qiagen |
| miR-181d primer | MS00031500 | Qiagen |

**Table S1.**

| **Gene** | **Forward primer** | **Reverse primer** |
| --- | --- | --- |
| GAPDH | GAGAGGCCCTATCCCAACTC | GTGGGTGCAGCGAACTTTAT |
| COL1A1 | TGACTGGAAGAGCGGAGAGT | CAGACGGCTGAGTAGGGAAC |
| COL3A1 | CTGTAACATGGAAACTGGGGAAA | CCATAGCTGAACTGAAAACCACC |
| COL5A1 | CCTGGCATCAACTTGTCCGATGG | GTGGTCACTGCGGCTGAGGAACTTC |
| COL5A2 | TGGGGACTGATGGTACACCT | GGATCACCCGATTGTCCTCG |
| COL12A1 | CCAGACGACCACGCTCAAT | TCTTCTCCATGACCGAAGTGG |
| PRELP | CCCACACCCAGATTTCCTCAG | TGGACAGTCAGGGAAGACAGA |

Table 2: List of used mRNA primers sequences

**Table S2.**
